# An Interplay between Senescence, Apoptosis and Autophagy in Glioblastoma Multiforme—Role in Pathogenesis and Therapeutic Perspective

**DOI:** 10.3390/ijms19030889

**Published:** 2018-03-17

**Authors:** Elzbieta Pawlowska, Joanna Szczepanska, Magdalena Szatkowska, Janusz Blasiak

**Affiliations:** 1 Department of Orthodontics, Medical University of Lodz, 92-216 Lodz, Poland; elzbieta.pawlowska@umed.lodz.pl; 2 Department of Pediatric Dentistry, Medical University of Lodz, 92-216 Lodz, Poland; joanna.szczepanska@umed.lodz.pl; 3 Department of Molecular Genetics, Faculty of Biology and Environmental Protection, University of Lodz, 90-236 Lodz, Poland; magdalena.szatkowska@biol.uni.lodz.pl

**Keywords:** glioblastoma, senescence, autophagy, apoptosis, temozolomide, DNA damage response

## Abstract

Autophagy, cellular senescence, programmed cell death and necrosis are key responses of a cell facing a stress. These effects are partly interconnected, but regulation of their mutual interactions is not completely clear. That regulation seems to be especially important in cancer cells, which have their own program of development and demand more nutrition and energy than normal cells. Glioblastoma multiforme (GBM) belongs to the most aggressive and most difficult to cure cancers, so studies on its pathogenesis and new therapeutic strategies are justified. Using an animal model, it was shown that autophagy is required for GBM development. Temozolomide (TMZ) is the key drug in GBM chemotherapy and it was reported to induce senescence, autophagy and apoptosis in GBM. In some GBM cells, TMZ induces small toxicity despite its significant concentration and GBM cells can be intrinsically resistant to apoptosis. Resveratrol, a natural compound, was shown to potentiate anticancer effect of TMZ in GBM cells through the abrogation G2-arrest and mitotic catastrophe resulting in senescence of GBM cells. Autophagy is the key player in TMZ resistance in GBM. TMZ can induce apoptosis due to selective inhibition of autophagy, in which autophagic vehicles accumulate as their fusion with lysosomes is blocked. Modulation of autophagic action of TMZ with autophagy inhibitors can result in opposite outcomes, depending on the step targeted in autophagic flux. Studies on relationships between senescence, autophagy and apoptosis can open new therapeutic perspectives in GBM.

## 1. Introduction

Cancer transformation is a complex phenomenon with a series of events initiated usually in a single cell and leading to a clinically detectable tumor. Many different aspects of cancer transformation and many features of cancer cells decide about cancer progression and tumor aggressiveness, but genomic instability is a feature of almost all, if not all, cancer cells [1]. Therefore, the functioning of a cancer cell in the state of genomic instability can be a key issue to understand mechanisms of pathogenesis of majority of cancers. Genomic instability is associated with malfunctions of DNA repair systems [2]. On the other hand, DNA repair is a major component of DNA damage response (DDR), a collection of functionally linked events initiated by a damage to cellular DNA [3]. DNA damage in a cell with dysregulated DDR can result in a mutation contributing to cancer transformation. When the extent of DNA damage excesses DNA repair potential, such as in the case of many anticancer drugs, a cell can be given, or gives itself, an additional time for that damage repair at the G1/S or G2/M checkpoint of the cell cycle or be directed on a programmed death pathway, usually apoptosis [4]. However, cancer cells can avoid this scenario and survive treatment with anticancer drugs due to deregulated DDR and apoptosis resistance. Therefore, apoptosis cannot be an optimal target in cancer therapy, but anti-apoptotic mechanisms can be important in cancer pathogenesis, as they strengthen and potentiate genomic instability.

Intensive proliferation of cancer cells due to their own program different from that in normal cells is their key feature and a target of many anticancer strategies primarily aimed at stopping cellular divisions. These strategies can be associated with apoptosis, but usually apoptosis per se is not their final goal. Cellular senescence, further referred to as senescence, is a cellular state characterized by several structural and functional features, including loss of mitotic activity resulting in cessation of cellular divisions. Therefore, to stop proliferation of cancer cells, it is not necessary to kill them all, instead induction of senescence pathway may be sufficient. Several senescence-inducing drugs are under clinical trials [5].

Cellular senescence is associated with the production of usually harmful cellular species, which can be cleared by autophagy, a process of removal of damaged or no longer needed cellular products [6]. In general, autophagy can help a cell to cope with many kinds of stress and cancer cells can use it to promote their intensive proliferation when their access to nutritional compounds is limited and to survive some therapies [7,8,9,10]. However, massive autophagy, which can lead to destructive cell “self-eating”, can induce apoptosis as suggested by the studies in which inhibition of autophagy decreased apoptosis [11]. “Autophagic cell death” is a term related to cellular death resulting from autophagy inhibition [12]. However, there are some open questions concerning autonomy of autophagic death effect [13]. Autophagic vehicles are present in many dying cells, which misleads to the conclusion that autophagy is a mediator of cell death. In fact, autophagic vacualization can help a cell survive against stress eventually causing its death. Despite many disputable and even conflicting results, autophagy may be important for the fate of cancer cells, especially when they face chemo- or radiotherapy. In addition, in multicellular organisms, autophagy-related death can be considered as a pro-survival mechanism, decreasing the number of cells in limited nutrient supply [14,15].

As mentioned, autophagy can clear cellular waste products resulted from senescence, but the detailed relationship between autophagy and senescence is not completely clear and there are contradictory data showing both that inhibition of autophagy can favor cell senescence and that autophagy is necessary for senescence [16,17]. This relationship is even more complex in cancer as there are reports suggesting both tumor-suppressive and tumor-promoting roles of autophagy as well as lack of its influence on cancer transformation [18]. Loss of the Atg family proteins is frequently associated with cancer induction and development, suggesting tumor suppressor-like role of autophagy in cancer [19]. It seems that p62/SQSTM1 (sequestome 1), an autophagy adaptive protein, can be important for autophagy in cancer [20]. It was shown that p62/SQSTM1 displayed oncogenic properties in hepatocellular carcinoma [21].

Therefore apoptosis, senescence and autophagy interconnect in cancer. They play a role both in cancer pathophysiology and response to anticancer therapy, so this subject can be important for both cancer biology and clinic. Glioblastoma (glioblastoma multiforme, GBM) belongs to the most difficult to cure and most aggressive cancers and is the most frequent brain cancer in humans. Despite that, its molecular pathogenesis is poorly known. Therefore, searching for molecular mechanisms underlying GBM pathogenesis, especially in the respect of its diagnosis, prognosis and therapy is needed and justified.

## 2. Glioblastoma Multiforme

GBM is the grade IV astrocytoma, a tumor that emerges from the glia. The aggressiveness of GBM and weak therapeutic potential against it causes most GBM patients to die within one year after diagnosis [22,23]. GBM is difficult to treat because it contains a heterogeneous population of cells characterized by a high genetic instability and tendency to occur anywhere in the brain [24]. Therefore, some types of cells from tumor population may positively respond to certain therapies, while others may not be affected at all [25].

**Figure 1:**
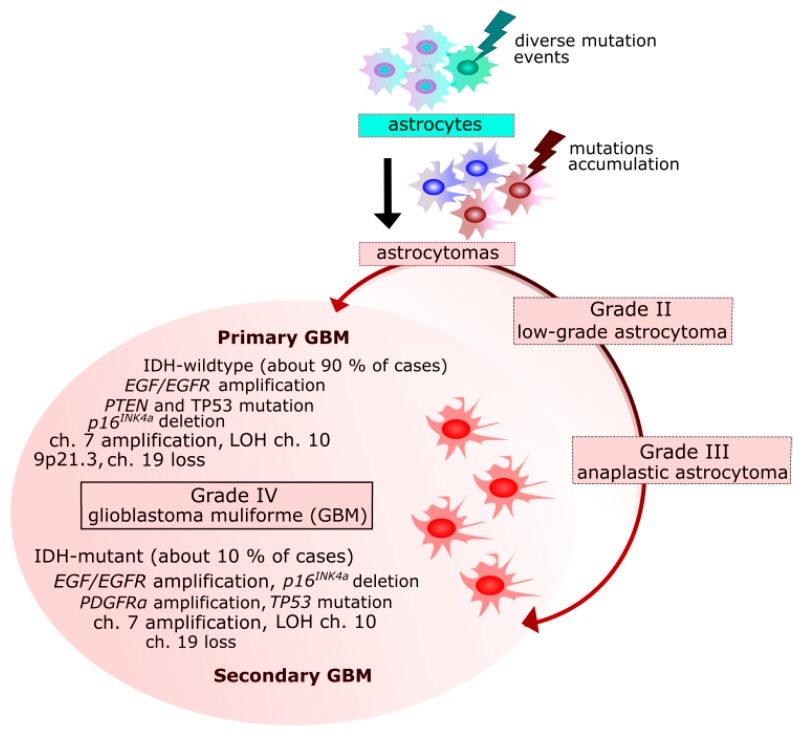
Primary and secondary glioblastoma multiforme (GBM)—origin and genetic changes. EGF/EGFR, epidermal growth factor/receptor; MDM2, mouse double minute 2 homolog; CDKN2A, cyclin dependent kinase inhibitor 2A; RB, retinoblastoma; TP53, tumor protein p53; PTEN, phosphatase and tensin homolog; NF1, neurofibromin 1; PDGFRα, platelet derived growth factor receptor alpha; IDH1/2, isocitrate dehydrogenase (NADP(+)) 1, cytosolic/2, mitochondrial; LOH, loss of heterozygosity; ch., chromosome(s).

GBM cases can be divided into primary and secondary tumors that are clinically indistinguishable, but present different molecular features [26] (Figure 1). Primary GBMs are characterized by more genetic alterations than their secondary counterparts [27,28]. Primary GBM arises de novo, without earlier histological evidence of a cancer precursor and represent over 90% of all GBM cases. These tumors occur mostly in the elderly, the mean age at diagnosis is 62, and half of patients die in less than three months. Secondary GBM arises from the progression of a lower grade glioma and is characterized by a moderate time course. These tumors are predominantly found in younger populations, the mean age at diagnosis is 45, and they represent a minority of all GBMs [27,28].

The Cancer Genome Atlas (TCGA) classifies GBMs into subtypes based on gene expression, genomic data, DNA mutation and gene copy. According to this classification, GBM is divided into four molecular subtypes, namely, classical, mesenchymal, proneural and neural, with distinct molecular features [29].

**Figure 2:**
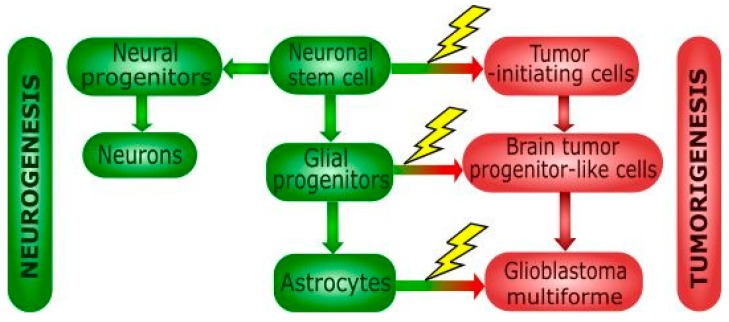
Differentiation of neural stem cells and cancer transformation. Neuronal stem cells can produce normal (green blocks) or cancer (red blocks) cells of various potential. Mutations occurring during differentiation of neural stem cell (NSCs) can contribute to tumor formation. NSCs can differentiate into neural/glial progenitor cell, but their transformation can lead to the formation of tumor-initiating cells (TICs). Neural progenitors undergo differentiation into neuronal cells, but glial progenitors differentiate into astrocytes. Genetic aberrations in glial progenitor cell can lead to tumor progenitor stem-like cells, but oligodendrocytes and astrocytes are also potential candidates involved in glioblastoma formation. TICs are also committed to the tumor formation via its differentiation into brain tumor progenitor-like cells.

Better understanding the origin of GBM cells was possible thanks to the discovery of multipotent neuronal stem cells (NSCs) in the brain. Normal brain cells, as astrocytes, are suggested to accommodate mutations inducing their dedifferentiation and acquiring cancer phenotype resulting in GBM stem cells [30]. During transit-amplifying phase, NSCs can undergo transformation events to the tumor-initiating cells (TICs) and tumor progenitor-like cells [31] (Figure 2).

Therapy of GBM is difficult and currently there is no treatment that could be considered as curative, so it is rather palliative and includes surgery, radiotherapy and chemotherapy with temozolomide (TMZ), a DNA alkylating agent [32]. In some GBM cells, TMZ induces extensive DNA damage, resulting in cell cycle arrest at the G2/M checkpoint to give the cell an additional time to repair damage to its DNA. This effect is mediated by the activation of the ATM/ATR-Chk1/2 (ataxia telangiectasia mutated/ataxia telangiectasia and Rad3-related-checkpoint kinase 1/2) axis, subsequent activation of the Wee1 kinase, Cdk1 (cyclin dependent kinase 1) activation and inhibition of CDC25A (cell division cycle 25A) [33,34]. This G2/M arrest can be considered as a prosurvival effect as otherwise the cell would enter mitosis with damaged DNA.

TMZ, approved by FDA in 2005, is the most widely and effective drug in GBM treatment in adults [35,36]. However, median time of the disease recurrence after standard therapy is about seven months [37]. Several other chemical agents and various regimes of radiotherapy are applied, but the results are still far from expectations. Low effectiveness of or even resistance to TMZ is another problem which will be discussed later.

## 3. Senescence in Glioblastoma

Senescent cells are featured by cell cycle arrest and inability to reinitiate the cycle unless transformed. In general, senescence can be divided into two classes: replicative senescence and premature senescence [38]. Replicative senescence is associated with the end replication problem—DNA polymerase is unable to initiate replication of the very ends of linear chromosomes, which leads to shortening of chromosome ends in each cellular division-chromosome erosion. When such erosion can affect genes important for normal functioning, a cell undergoes replicative senescence and stops division. The checkpoint for replicative senescence is at the G1/S interface of the cell cycle [39]. Premature senescence is induced by various cellular stresses, including oxidative, genotoxic and replicative stress and is not associated with telomere erosion [40].

Cancer cells circumvent end replication problem by several mechanisms, mainly by the activation of telomerase, a reverse transcriptase using an RNA template to initiate replication of chromosome ends or alternate lengthening of telomeres (ALT), a complex mechanism, which can be different for different cancer cells [41]. Additionally, telomeric DNA is covered by the six-protein complex called shelterin (telosome) [42]. This complex acts protectively against telomere erosion and regulates the activity of telomerase and therefore can play a role in cancer transformation [43].

**Figure 3:**
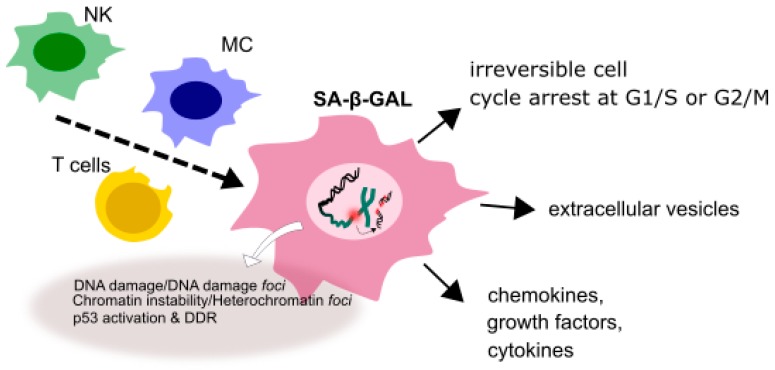
A senescent cell displaying senescence-associated phenotype (SASP). In a senescent state a cell is irreversibly stopped at the G1/S or G2/M checkpoint and shows different morphology than its normal counterpart. It displays increased activity of senescence-associated-β-galactosidase (SA-β-gal), releases various soluble factor, enhanced extent of DNA damage and chromosomal aberrations collectively determining SASP.

Besides replication arrest, senescent cells have several other specific morphological and biochemical features, including expanded cytoplasm and enhanced cytoplasmic granularity as well as increased activity of senescence-associated β-galactosidase (SA-β-gal). They also display elevated level of DNA damage and chromosomal aberrations. These and other characteristics determine the senescence-associated secretory phenotype (SASP) typical for a senescent cell (Figure 3). Cells exhibiting SASP release chemokines, cytokines, growth factors, extracellular vehicles and other soluble factors and can be targeted and cleared by the components of immunological system, including macrophages, NK cells and T-lymphocytes, which can, in turn, lead to a low-grade inflammation contributing to aging of an organism (inflammaging) [44]. Repeated or chronic exposure to a stress can result in the accumulation of prematurely senescent cells with age and contribute to tissue aging [45].

Although SASP has a high specificity to senescent cells, not a single marker or even a single set of markers may be applied to conclusively identify a senescent cell. This probably follows from a complex relationship between cellular senescence and aging of tissues, organs and organisms [46].

Senescence in cancer cells presents a serious problem as a cell, which irreversibly lost its ability to divide is in fact no longer a cancer cell per se. However, senescent cells are considered to have pro-cancerogenic properties [47]. It seems important that senescent cells can display resistance to apoptosis [48]. On the other hand, senescence with permanently arrested cellular division, can be, similar to apoptosis, an alternative pathway to avoid cancer transformation. Premature senescence can be induced by oncogenes, which supports its anti-tumorigenic potential [49]. Senescence studies in glioblastoma are additionally complicated by the observation that primary GBM cells isolated from patients behave differently than glioblastoma cell lines and the presence of serum can be critical for their properties, including tumorigenic potential and telomerase activity [50,51].

Glioma cells can avoid replicative senescence resulting from mutations in the promoter of the *TERT* (telomerase reverse transcriptase) gene [52,53]. In addition, mutations in genes encoding shelterin proteins were observed in glioma cases [54].

Studies in glioblastoma cell lines showed that premature senescence in these cells can be induced in a p53-dependent and -independent fashion [55,56,57,58]. Several proteins important for GBM cell genesis can be linked with senescence. It was shown that Forkhead Box O1 (FOXO1), a protein involved in cell cycle regulation and epithelial mesenchymal transition, could facilitate senescence by modulation of the expression of sirtuin 1 (SIRT1), a histone deacetylase [59]. However, SIRT1 also stimulates autophagy by deacetylation of essential autophagy proteins in many cancers (reviewed in) [60,61]. However, SIRT1 can be treated with a skepticism as a candidate for a leading protein in the cross-talk between senescence and autophagy in GBM, as it is a general function protein with no specificity or special affinity to gliomas.

It seems that many pathways can be involved in senescence induction in GBM cells. It was reported that copper evoked premature senescence in the GBM U87-MG cells with concomitant downregulation of the BMI1 (proto-oncogene, polycomb ring finger, B lymphoma Mo-MLV insertion region 1 homolog (mouse)) pathway [62]. BMI1 was shown to be involved in autophagy regulation in several cancers, including chronic myeloid leukemia, breast and ovarian cancers [63,64,65]. Research performed on the GBM U87-MG cell line, both wild-type and p53-mutated, showed that arsenite evoked premature senescence as a result of DNA damage in a p53/p21-depedent fashion [66]. Again, the p53/p21 pathway can be involved in many processes, especially associated with DNA damage and cannot be rather specifically attributed to GBM. It was shown that 14-3-3β, a scaffold protein, the expression of which correlates with malignance grade in astrocytomas, negatively regulated senescence in the GBM A172 cells through the ERK-SKP2-p27 (extracellular signal regulated kinase-S-phase kinase-associated protein 2-p27) pathway [67]. Another ERK-related pathway, which can be modulated in senescent GBM cells was reported by Liu et al. who showed that berberine, an isoquinoline alkaloid, induced senescence in downregulated EFGR-MEK-ERK (epidermal growth factor receptor-mitogen-activated protein kinase kinase-ERK) signaling pathway [68].

Nuclear hormone receptors REV-ERBα (NR1D1) and REV-ERBβ (NR1D2) are essential components of the circadian clock [69,70]. Sulii et al. showed that agonist of these REV-ERBs are lethal for cancer and oncogene-induced senescent cells and practically non-toxic for normal cells [71]. They were shown to inhibit glioblastoma growth in mice and NRD1 expression was positively correlated with survival of brain cancer patients. It was proposed that observed effects resulting from REV-ERBs modulation follows from the inactivation of lipogenesis and autophagy. Therefore, the relationship between senescence and autophagy can be important in pharmacological regulation of circadian mechanisms in GBM therapy.

Paget et al. showed that the depletion of the protein kinase iota (PKCι), a protein involved in neuronal plasticity and survival, evoked senescence in GBM cells without DDR activation [56]. In their subsequent study, these authors showed that senescent GBM cells displayed aberrant structure of centromeres, were polyploid and arrested at the G1/S checkpoint, which suggested mitotic slippage, a premature exit of a cell from mitosis into G1 phase [58]. Therefore, modulation of the PKCι protein can be important for mitotic slippage-induced senescence of GBM cells.

## 4. Autophagy in Glioblastoma

**Figure 4:**
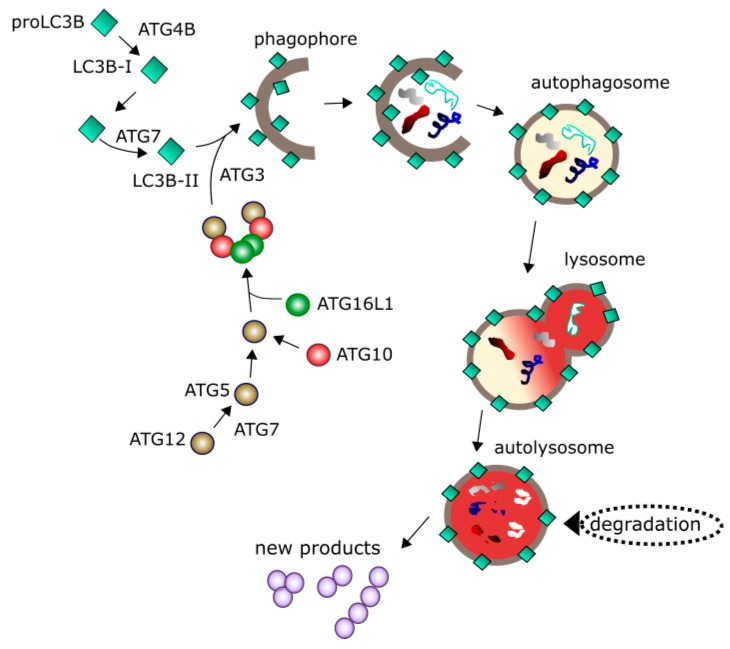
Autophagy is initiated by a series of events leading to the nucleation of the phagophore, a structure sequestering material to be degraded (cargo) with the involvement of many proteins including a palette of the ATG (autophagy related) proteins 7, 2 variants of LC3B (microtubule associated protein 1 light chain 3 beta). Maturation of phagophore results in the formation of a double-membrane vehicle (autophagosome) encircling the cargo. Autophagosome fuses with lysosome into autolysosme, in which degradation of the cargo occurs. Degraded material can be recycled providing new products to be used by the cell. Many other proteins regulating autophagy are not presented here.

During autophagy, damaged or no longer needed material (cargo) is encapsulated in series of double-membrane vesicles and targeted to lysosmal degradation (Figure 4). Autophagy can be cellular response to nutrient deprivation and is then associated with degradation of cellular components and subsequent recycling of degraded cargo to produce amino acids or energy-rich biomolecules. This process requires many proteins and protein complexes to form phagophore, a double-membrane structure encapsulating the cargo and resulting in autophagosome [6]. Autophagosome must mature to fuse with lysosome, where degradation occurs. This maturation is supported by ubiquitin-like proteins, including MAP1LC3/LC3 (microtubule associated protein 1 light chain 3). Growing phagophore recruits cytosolic LC3 (LC3-I), which is conjugated with phosphatidylethanolamine to form LC-3II in a reaction catalyzed by the ATG3, ATG7 proteins and the ATG12-ATG5-ATG16L1 complex. Activation of autophagy occurs with the involvement of various other proteins, including ULK1 (Unc-51 like autophagy activating kinase) kinase, ATG13 and RB1CC1/FIP200 (RB1-inducible coiled-coil 1).

Autophagy is reported to play a role in the pathogenesis of many cancers and therefore it can be considered as a target in cancer therapy [72,73,74]. However, similarly to senescence, it is difficult to unequivocally determine the role of autophagy in cancer transformation. Tumor growth can be supported by providing necessary nutrients by autophagosomal recycling of wasted material. On the other hand, cancer transformation can be prevented by autophagic clearing carcinogenic cellular waste.

Studies performed on tumors obtained from GBM patients after operation and undergoing radiotherapy, as well as on GBM cell lines, showed changes in the pattern of expression of several proteins important for autophagy, including C3A (complement C3), LC3B, p62, Beclin 1, ULK1 and ULK2 [75]. Some other proteins, which can play a regulatory role in the expression of autophagy-related proteins, including TFEB (transcription factor EB), PTEN (phosphatase and tensin homolog), Cathepsin D, HIF1α (hypoxia-inducible factor 1alpha), displayed different expression patterns from controls. The LC3 proteins have a distinct pattern of immunohistochemical staining in solid tumors, referred to as staining of “stone-like” structures (SLSs), which correlates with bad prognosis in cancer [76]. The appearance of SLS was associated with poor prognosis in GBM patients undergoing radiotherapy. No other parameter was directly linked with the survival rate of post-irradiated patients.

It was shown with an animal model of KRAS-dependent gliomagenesis that autophagy is required for GBM development [77]. It was underlined in that study that studies on GBM cell lines are performed in a particular stage of carcinogenesis, so they cannot provide information on how a particular process influenced cancer transformation since its initiation. Following this idea, Gammoth et al. constructed a mouse model with inhibited autophagy and activated oncogene—the mouse had a virus-derived KRAS activation and shRNA ATG7-mediated autophagy inhibition. They observed a lack of clonogenic growth in low oxygen conditions, which was attributed to a lower extent of phosphorylation of the AKT (AKT serine/threonine kinase) and MAPK1/3 (mitogen-activated protein kinase 1/3) proteins, indicating a compromised growth signaling in those conditions, when autophagy was inhibited. Moreover, the MTORC1 (mechanistic target of rapamycin complex 1) signaling seemed not to be indispensable. In turn, low-serum conditions induced senescence in the KRAS/shATG7 cells suggesting that cells are prone to senescence when autophagy is impaired. As no activation of p53 was observed in those conditions, senescence might be independent of this protein. Autophagy can support gliomagenesis through increased survival of cancer cells in unfavorable conditions, including growth-restrictive states. Of course, translation of these animal model-based studies into in vivo human gliomagenesis needs further research.

As mentioned GBM patients are characterized by defects in many signaling pathways underlined by mutations in important components of these pathways. A gain-of-function mutation was reported to occur in the receptor tyrosine kinase (RTK)-RAS class I phosphoinositide 3-kinase (PIK3) oncogenic pathway, which is related to autophagy [78,79]. It was observed that GBM tumors acquired resistance to antiangiogenic therapy by the induction of autophagy resulting from hypoxia [80]. This protective role of autophagy in GBM can be blocked by inactivation or deletion of tumor suppressor genes [81].

The regulation of autophagy is controlled by signaling pathways that also regulate tumorigenesis. Inactivation or deletion tumor suppressor genes, whose products are frequently associated with regulation of autophagy in tumor, may have impact on blocking the autophagy’s protective function in GBM. Deficiency in the expression of autophagy-regulating genes, Atg4C, Bif-1, Atg5 as well as Beclin 1 and frequent dysregulation of the PIK3-AKT-mTOR signaling pathway are accompanied with GBM development [82].

Suberoylanilide hydroxamic acid (SAHA) is a histone deacetylase inhibitor that can change the chromatin structure and, in this way, modulate DDR [83]. Therefore, it is considered in cancer therapy and in fact it has been under several clinical trials [84]. Chiao et al. reported that SAHA specifically induced autophagy and promoted glioblastoma stem cells (GSCs) death by apoptosis both in vitro and in vivo [85]. Autophagy was activated by the downregulation of the AKT-mTOR pathway. When autophagy was inhibited or depleted, SAHA induced apoptosis, again confirming that autophagy can be pro-survival reaction of cancer cells facing therapy-associated stress. Moreover, chemical inhibitors of autophagy can synergize with SAHA in induction of apoptotic effects in GSCs, which can be exploited in GBM therapy. Moreover, non-apoptotic doses of SAHA induced senescence in GSCs. Therefore, SAHA provides another example of a delicate interbalance within the senescence–autophagy–apoptosis triad in GBM cells and its significance in GBM treatment.

It was shown that inhibition of the PIK3 and mTOR signaling pathways activated autophagy in GBM cells [86]. Moreover, that study showed that inhibition of mTORC1/2 complexes additively induced autophagy and inhibition of autophagosome formation in the presence of rapamycin did not induce apoptosis. However, apoptosis was promoted by a combined action of rapamycin with inhibitors of autophagosome maturation and PI3K. Therefore, chemicals used to modify the signaling pathway important in autophagy and apoptosis have a therapeutic potential in GBM.

## 5. Therapeutic Potential

**Figure 5:**
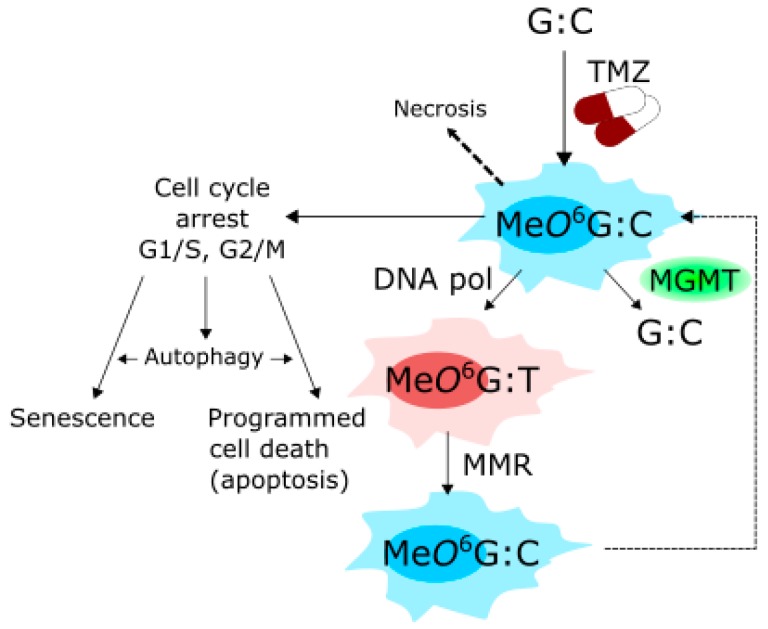
Mechanism of action of temolozamide (TMZ). TMZ methylates guanine (G) paired with cytosine (C) in the genomic DNA at the *O*^6^ position, yielding *O*^6^-methyl guanine (Me*O*^6^G). The methyl group can be removed from G by *O*^6^-methylguanine-DNA methyltransferase (MGMT), otherwise Me*O*^6^G can be paired with thymine (T) by DNA polymerase (DNA pol) in the next replication round, giving the Me*O*^6^G:T mismatch, which can be processed by mismatch repair (MMR) system. When these DNA repair mechanisms cannot cope with all Me*O*^6^Gs and their consequences, the cell is arrested at the cell cycle checkpoint, G1/S or G2/M, to have more time for repair. If this fails, the cell can adopt senescence or be directed on a programmed death pathway, usually apoptosis. Autophagy is another effect, which can be induced by TMZ and it can interact with senescence and apoptosis (more details in the main text). Extremely high TMZ concentrations can induce necrosis due to general toxicity of this drug. Me*O*^6^G is not the only DNA damage induced by TMZ.

As mentioned, TMZ is the main drug applied in GBM chemotherapy. It is an alkylating imidazotetrazine, which methylates guanine in the genomic DNA at the *O*^6^ position (Figure 5). This methylated guanine can mismatch with thymine in the next replication cycle. The G:T mispairing can be repaired by mismatch repair (MMR), a DNA repair system [87,88]. Methylated guanine can also be directly demethylated by *O*^6^-methylguanine-DNA methyltransferase (MGMT), which removes methyl group from DNA, binding it to one of its residues [89]. However, many glioma cell lines and primary glioma cells are reported to be MGTM defective [90]. Therefore, functioning of these DNA repair systems can be important for the efficacy of GBM therapy with TMZ and resistance to this drug, which was reported in several studies (reviewed in [82]). However, if the extent of DNA damage exceeds cell capacity to repair it, the cell is arrested at a cell cycle checkpoint, usually G1/S or G2/M, and given the additional time needed to completely repair DNA damage. If this falls, different scenarios are possible depending on many factors, including the extent of unrepaired DNA damage and current cellular conditions. The cell can activate a programmed death pathway or stop cellular division, transiently (quiescence) or permanently (senescence). Therefore, the mechanism of anticancer action of TMZ can be a combination of several mechanisms. At extremely high concentrations TMZ can induce necrosis, which is likely associated with its general toxicity. Other scenarios are also possible, as Me*O*^6^G is not the only DNA damage, which can be induced by TMZ.

TMZ was reported to induce senescence, autophagy and apoptosis in GBM cells [91,92,93]. Therefore, the cross-talk between senescence, autophagy and apoptosis is important for GBM chemotherapy with TMZ, especially that GBM cells can be intrinsically resistant to apoptosis. In this context, searching for compounds that potentiate senescent and/or autophagic pathways in TMZ action is needed. Filippi-Chela et al. showed that resveratrol, a natural compound which adds to TMZ toxicity in GBM cells both in vitro and in vivo, abrogated TMZ-induced G2-arrest and forced cells into mitosis, which led to mitotic catastrophe (MC), resulting in senescence and inhibition of clonogenic activity of GBM cells [94]. Therefore, senescence is the key event in enforcing anticancer action of TMZ in GBM cells by resveratrol. These studies also showed that autophagy was not involved in the potentiation of TMZ toxic effect due to resveratrol co-treatment, but played rather a protective role.

TMZ singly frequently induces too low toxicity in GBM cells to rich clinical significance and some cells display resistance to this compound. Although, as mentioned, some GBM cells are naturally resistant to apoptosis, breaking apoptosis resistance is not the only way to sensitize GBM cells to TMZ. Thalidomide, an immunomodulatory drug, was shown to enhance the toxicity of TMZ by affecting the PI3K-Akt-mTOR pathway, which is important in autophagy [95]. However, it was shown that TMZ induced a sustained inhibition of Akt/mTOR, which in turn resulted in a transient induction of autophagy and finally to resistance of GBM cells to TMZ therapy [96]. As mentioned, autophagy can be involved in both pro-survival and pro-death mechanisms in cancer cells, the former often contributing to the resistance to therapy. Autophagy induced by TMZ can function as a pro-survival mechanism as its induction led to an increase in apoptosis level in GBM upon TMZ treatment [97,98]. Therefore, inhibition of autophagy prior to TMZ administration is a strategy to fight TMZ resistance. Chloroquine (CQ), an autophagy inhibitor, and its analogs, have been tested in combination with TMZ in clinical trials in gliomas and other cancers [99,100,101,102]. However, it seems that combined action of TMZ and CQ or its analogs depends on the cellular context, in which the p53 protein can play a pivotal role [103]. Indeed, p53 status is important for the action of TMZ itself [92].

Modulation of autophagy at its different steps can have different outcomes for TMZ treatment, as shown by Kanzawa et al. [93]. In practice, the use of different autophagy inhibitors, can results in different behavior of GBM cells. 3-MA (3-methyladenine) inhibits the localization of LC3 to autophagosome membrane and increases survival of GBM cells, but bafilomycin A1 inhibits autophagy, without affecting LC3 localization. When autophagic vehicles accumulate due to their inability to fuse with lysosome, this lead to permeabilization of lysosmal and mitochondrial membranes resulting in apoptosis. Therefore, although apoptosis is the final outcome of TMZ action, autophagy mechanistically determines efficacy of TMZ treatment.

The complex relationship between senescence, autophagy and apoptosis in relation to TMZ therapy of GBM was addressed by Knizhnik et al. [97]. They assumed that these cellular responses as well as necrosis, can be switched by DNA damage induced by TMZ and tried to determine the type of such damage, which can be attributed to a particular response. They observed that TMZ induced senescence, autophagy and apoptosis in a time-dependent manner, specific for each response. When glioma isogenic cells expressed MGTM, the effect was abrogated, suggesting that *O*^6^MeG can trigger all these responses. Next, they observed that autophagy induced by *O*^6^MeG required MMR and ATM, a crucial DDR protein, involved in DNA double-strand break repair signaling and was reduced by homologous recombination. They showed that autophagy was a pro-survival mechanism and preceded apoptosis. In turn, cellular senescence also preceded apoptosis and was reduced when autophagy was revoked. The final conclusion was that autophagy induced by *O*^6^MeG resulted from TMZ action is a pro-survival mechanism, which directs the GBM cells on senescence pathway to avoid apoptosis. Necrosis was induced at a low level in that study. This important work showed not only fundamental properties of TMZ interaction, important for GBM therapy, but also thrown light on the role of autophagy in DDR, especially the interplay between autophagy and DNA repair, which is still poorly known [74].

As mentioned in the Introduction section, senescence and not apoptosis can be an ultimate target in cancer therapy. However, lastly cited works showed that senescence is a specific pro-survival mechanism of GBM cells to escape apoptosis. Therefore, there is apparent paradox: senescent therapy directs GBM cells into a state that is turned on to survive other anticancer treatment. There is no doubt that senescence is “better” than apoptosis from GBM cells point of view, but this is not necessarily the case from the therapeutic side. Hypothetically, senescent cells may represent non-invasive carcinoma in situ, although they release some substances, associated with SASP, which may be toxic for them and surrounding cells, but the anatomical relationships within cancer-affected organ or tissue are not changed due to senescence induction. When massive apoptosis occurs, cells may not release or leave toxic waste, but disappearing of a great number of cells from a specific location may result in serious changes in morphology and anatomy.

Although reactive oxygen species (ROS) are involved in pathogenesis of many diseases, including various cancers, their role in malignant transformation is somehow specific as cancer stem cells seem to have different ROS-related characteristic than normal cells [104]. This led to the concept of ROS-based anticancer therapy, which aims to substantially increase the amount of ROS in cancer cells, which cannot stand any longer and died [105]. However, again, the kind of ROS-induced death can be different for different conditions. Moreover, ROS can induce senescence and senescent cells can display a different reaction to ROS than proliferating cancer cells. Autophagy is another problem, as it can be induced by ROS. Similar to functions that can be attributed to autophagy in cancer, ROS can display at least two faces in cancer transformation—promotive and suppressive [104]. Therefore, the ROS–senescence–autophagy–cancer axis is quite complex and many outputs are possible. Moreover, normal cells are also sensitive to ROS and can die in result of their action. Despite this, ROS-based therapy is developed also in GBM. In fact, radiotherapy, which usually accompanies surgical resection and chemotherapy in GBM, is based on water radiolysis induced by ionizing radiation and overproduction of ROS, which damage biomolecules, including DNA, proteins and lipids. It was shown that verapamil, a classical drug used in diseases of nervous system, increased senescence induced by radiation in GBM cells by a decrease in intracellular ROS and calcium levels [106]. This effect was likely to disturbances of one or several signaling pathways in GBM cells due to deregulation of intracellular ROS level.

## 6. Conclusions and Perspectives

Studies with resveratrol and other compounds administrated together with TMZ justify further studies on development of drugs, which induce mitotic catastrophe after TMZ action, resulting in senescence of GBM cells [94]. The pro-survival mechanism of autophagy seems to be mainly responsible for therapeutic resistance of GBM cells to TMZ.

Two faces of autophagy in response to TMZ and other anticancer drugs can be considered, pro-survival and pro-death, resulting in the opposite effects, TMZ resistance and TMZ enhanced sensitivity, respectively. Therefore, it is important to establish the threshold dose of TMZ to choose between these effects. However, this dose can depend on many factors, including cell kind, cellular context and chemicals used to modulate TMZ action.

Although we focused on interrelationships among senescence, autophagy and apoptosis, necrosis should also be considered, not only as the extension of the senescence–autophagy–apoptosis axis, but also as an output of cancer therapy, which can be linked with different consequences for patients than apoptosis- or senescence-oriented therapy. Moreover, necroptosis, a kind of necrosis, which can be considered as its programmed variant, was reported to be induced through the inhibition of BMI1 [63]. However, this inhibition was mediated by autophagy, as BMI1 can be related to autophagy in many tumors, as mentioned earlier. Induction of necroptotic cell death can be a strategy in cancer therapy and be useful in breaking chemoresistance of cancer cells and is worth checking in GBM.

As autophagy can delay or even block apoptosis in GBM cells, it is a promising therapeutic target in this disease. Besides pharmacological modulation of autophagy in GBM, oncolytic adenoviruses, which induce extensive autophagy in glioma cells, have been tested [107,108]. It is especially interesting in the context of this review, as autophagy-inducing oncolytic viruses can be activated by the telomerase promoter, so they might be associated with senescence in glioma cells [109]. In general, modulation of anticancer immune response by autophagy opens new promising perspective in GBM therapy [110].

Therapy with autophagy-induced drugs present a kind of Trojan Horse, as stated by Lefranc and Kiss, as it can rescue cancer cells from apoptotic death, but instead “autophagic death” can be induced [111].

**Figure 6:**
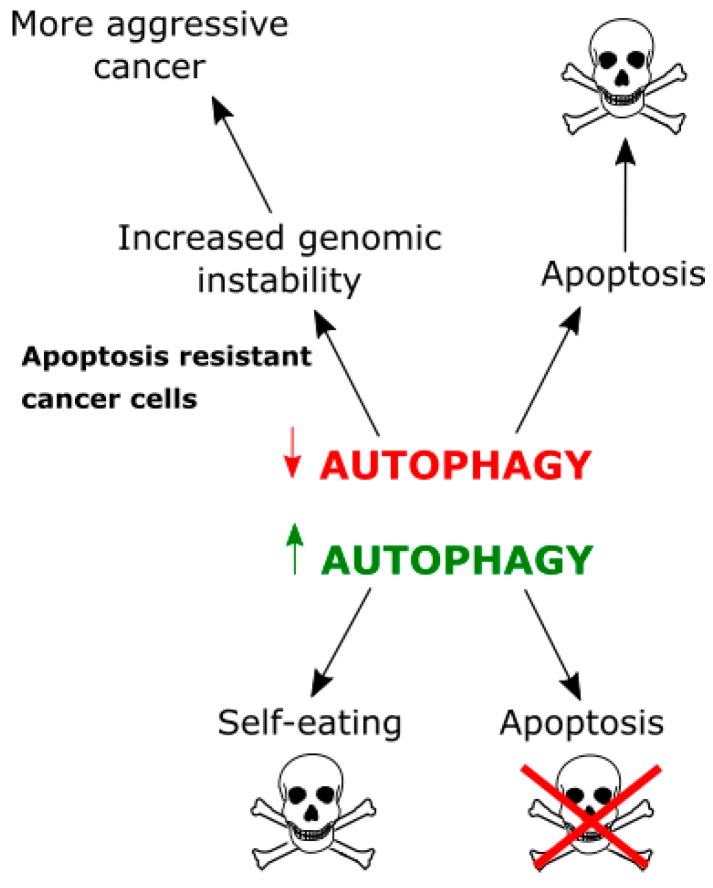
Relationships between autophagy and apoptosis in cancer cells. Autophagy can protect against apoptotic death, but, when too extensive, can lead to self-destruction of a cancer cell. On the other hand, inhibition of autophagy can result in apoptosis activation and cell death, but, when a cancer cell is intrinsically resistant to apoptosis, accumulation of toxic waste not cleared by autophagy can increase genomic instability and result in a more aggressive cancer.

Therefore, autophagy can be therapeutically targeted in at least two ways (Figure 6). First, it can be stimulated to the level, at which cancer cell will self-eats [112]. Second, it could be inhibited, not to block apoptosis [10]. However, many GBM cells are intrinsically resistant to apoptosis and inhibiting autophagy in these cells can lead to accumulation of DNA damage in GBM cells, without apoptosis induction, making them even more aggressive.

There are other drugs than TMZ applied with different efficacy in GBM therapy. First is bevacizumab (Avastin), a drug which affects angiogenesis and inhibits the progression of GBM, but it was not reported to improve overall survival of GBM patients. Of course, inhibition of angiogenesis, a “natural” process in neoplastic tumors, leads to a stress-induced reaction in cancer cells, which may take a form of apoptosis [113]. Bevacizumab can also induce autophagy, but autophagy can both promote and inhibit its action as well as the action of other anti-angiogenic drugs [114]. However, emerging evidence suggests a beneficial effect of autophagy inhibition in bevacizumab-based therapy in cancer, including GBM [114,115,116,117,118]. Carmustine, another drug used in GBM therapy, is an alkylating agent similar to TMZ [119]. However, its mechanism of anticancer action is different from TMZ as it dialkylates DNA and induces interstrand cross-links, which are serious DNA damage preventing DNA strand separation required for cell cycle progression, replication, transcription and recombination [120]. Carmustine also carbamoylates proteins, including DNA repair enzymes. All these effects suggest that carmustine can, directly or indirectly, induce senescence and autophagy, which was experimentally verified [106,121,122,123].

When we look for a relationship between senescence and autophagy, we should consider that most studies are performed on cell population, consisting of millions of cells and, at a given moment, different sub-populations can be in different states. Therefore, the time order of senescence, autophagy and apoptosis, which was reported by Knizhnik et al., provided the average value of specific parameters observed for a heterogeneous cell population [97]. Analogous situation in a single cell can be different and Filippi-Chela et al. postulated that the correlation between autophagy and senescence could not exist at all at the single cell level [96]. Moreover, they observed rather not generally accepted chain of events in GB

M cell after TMZ treatment, in which autophagy was followed by senescence. Therefore, single cell-based research may open a new senescence- and autophagy-related therapeutic perspective in GBM.
